# Minimal Solution of Singular LR Fuzzy Linear Systems

**DOI:** 10.1155/2014/517218

**Published:** 2014-03-11

**Authors:** M. Nikuie, M. Z. Ahmad

**Affiliations:** ^1^Sama Technical and Vocational Training College, Islamic Azad University, Urmia Branch, Urmia, Iran; ^2^Institute of Engineering Mathematics, Universiti Malaysia Perlis, Pauh Putra Main Campus, 02600 Arau, Perlis, Malaysia

## Abstract

In this paper, the singular LR fuzzy linear system is introduced. Such systems are divided into two parts: singular consistent LR fuzzy linear systems and singular inconsistent LR fuzzy linear systems. The capability of the generalized inverses such as Drazin inverse, pseudoinverse, and {1}-inverse in finding minimal solution of singular consistent LR fuzzy linear systems is investigated.

## 1. Introduction

The concept of fuzzy numbers and fuzzy arithmetic operations was first introduced and investigated by Zadeh [1, 2], Dubois and Prade [3], and Nahmias [4]. Some different approaches to fuzzy numbers and the structure of fuzzy number spaces were given by Puri and Ralescu [5], Goetschel and Voxman [6, 7], and Cong-Xin and Ming [8, 9]. Fuzzy systems are used to a variety of problems ranging from control chaotic systems [10] to fuzzy metric spaces [11], fuzzy linear systems, fuzzy differential equations [12], particle physics [13], and so on. System of simulations linear equations plays major role in various areas such as mathematics, physics, statistics, engineering, and social sciences [14]. Linear systems of equations plays major role in various areas of sciences and engineering such as fuzzy mathematics, and so forth [15]. The linear system of equations

(1) $$\begin{array}{c} A\tilde{x}=\tilde{b}, \end{array}$$

where *A* is a *n* × *n* crisp matrix and the right-hand side is a vector of fuzzy numbers in parametric form, is called a fuzzy linear system (FLS). Friedman et al. [14] use an embedding method for solving a special case of (1) when *A* is a nonsingular matrix. They introduce new notions for solution of a FLS (1) and transform the FLS to a crisp linear system. In their method, the ordinary inverse matrix is used to solve the FLS. The fuzzy linear system (1) where *A* is a *n* × *n* singular crisp matrix and the right-hand side is a vector of fuzzy numbers in parametric form is called a singular fuzzy linear system (SFLS) [16]. The capability of pseudoinverse [17] and Drazin inverse [16] in solving fuzzy linear systems and {1}-inverse [18] and {1,3}-inverse [19] in finding fuzzy least-square solutions of inconsistent fuzzy linear systems has been studied.

Ordinary inverse for any *n* × *n* nonsingular matrix exists and is unique. Unlike the case of the nonsingular matrix, which has a single unique inverse for all purposes, there are different generalized inverses for different purposes. For some purposes, as in the examples of solutions of linear systems, there is not a unique inverse, but any matrix of a certain class will do. Generalized inverses have various applications in the theory of finite Markov chains, the study of singular differentia and difference equations, the investigation of Cesaro-Neumann iterations, cryptograph, iterative methods in numerical analysis, multibody system dynamics, and others [20–23].

The fuzzy linear system (1) with LR fuzzy variables and LR fuzzy right-hand side is called a fuzzy LR linear system (FLRLS). Ghanbari et al. [24, 25] give the exact and approximated solutions of fuzzy LR linear systems. They give new algorithms using a least-squares model and the ABS approach. LR-type fuzzy systems of linear algebraic equations are discussed by Abramovich et al. in [26], and based on their work in [27] Allahviranloo et al. study LR fuzzy linear systems. They prove existence of fuzzy number vector solution of LR fuzzy linear system by solving optimization problem and obtain the approximated and exact solutions of mentioned systems. This approximated solution in general is different from the weak fuzzy solution considered by Friedman et al. [14, 25].

Here, the singular LR fuzzy linear systems are introduced. Such systems are divided into two parts: singular consistent LR fuzzy linear systems and singular inconsistent LR fuzzy linear systems. In this paper, the capability of the generalized inverses in solving singular consistent LR fuzzy linear systems is investigated. In Section 2, we recall some preliminaries. The purpose of this paper is to propose a method for solving singular LR fuzzy linear systems on the LR fuzzy linear systems discussed in Section 3. The proposed method is given in Section 4. Numerical examples are given in Section 5 followed by suggestions and concluding remarks in Section 6.

## 2. Preliminaries

This section gives a brief summary of index of matrix, Drazin inverse, and LR fuzzy numbers. We refer the reader to [28–31] for more information.

**Definition 1**

Let *A* ∈ *C* ^*n*×*n*^. The index of *A* is the size of the largest Jordan block corresponding to the zero eigenvalue of *A* and is denoted by ind(*A*).

**Definition 2**

Let *A* ∈ *C* ^*n*×*n*^, with ind(*A*) = *k*. The matrix *X* of order *n* is the Drazin inverse of *A*, denoted by *A* ^*D*^, if *X* satisfies the following conditions:

(2) $$\begin{array}{c} AX=XA,\;\;XAX=X,\;\;A^{k}XA=A^{k}. \end{array}$$

**Definition 3**

Let *A* ∈ *C* ^*m*×*n*^. The matrix *X* of order *n* × *m* is the pseudoinverse of *A*, denoted by *A* ^+^, if *X* satisfies the following conditions:

(3) $$\begin{array}{c} AXA=A,\;\;XAX=X,\\ {\left(AX\right)}^{*}=AX,\;\;{\left(XA\right)}^{*}=XA. \end{array}$$

**Definition 4**

Let *A* ∈ *C* ^*m*×*n*^. The matrix *X* of order *n* × *m* is the {1}-inverse of *A*, denoted by *A* ^(1)^, if *X* satisfies the following condition:

(4) $$\begin{array}{c} AXA=A. \end{array}$$

**Definition 5**

The fuzzy number $\tilde{A}$ is of LR-type if there exist shape functions *L* (for left), *R* (for right), and scalers *α* > 0, *β* > 0 with

(5) $$\begin{array}{c} \mu_{\tilde{A}}\left(x\right)=\{\begin{array}{cc} L\left(\frac{a-x}{\alpha }\right), & x\leq a,\\ R\left(\frac{x-a}{\beta }\right), & x\geq a. \end{array} \end{array}$$

The mean value of $\tilde{A}$, *a*, is a real number and *α*, *β* are called the left and right spreads, respectively. $\tilde{A}$ is denoted by (*a*, *α*, *β*)_LR_. A crisp number *a* is specified to be (*a*, 0,0)_LR_.

**Definition 6**

The fuzzy number $\tilde{A}={\left(m,\alpha ,\alpha \right)}_{LL}$ is a symmetric fuzzy number and is denoted by $\tilde{A}={\left(m,\alpha \right)}_{L}$.

**Definition 7**

The set of LR fuzzy numbers is denoted by *F*(*R* ^1^)_LR_. For arbitrary LR fuzzy numbers $\tilde{M}={\left(m,\alpha ,\beta \right)}_{\text{LR}}$, $\tilde{N}={\left(n,\gamma ,\delta \right)}_{\text{LR}}$, and *λ* ∈ *R* ^+^, we have

1. $\lambda \tilde{M}={\left(\lambda m,\lambda \alpha ,\lambda \beta \right)}_{\text{LR}}$,
$-\tilde{M}={\left(-m,\beta ,\alpha \right)}_{\text{LR}}$,
3. $\tilde{M}⊕\tilde{N}={\left(m+n,\alpha +\gamma ,\beta +\delta \right)}_{\text{LR}}$.

## 3. Singular LR Fuzzy Linear Systems

In this section, singular LR fuzzy linear systems are introduced and the existing methods for solving LR fuzzy linear systems are investigated.

### 3.1. Shortcomings of the Existing Methods

In this subsection, the shortcomings of the existing methods for solving LR fuzzy linear systems are pointed out.

(1) Ghanbari and Mahdavi-Amiri [24] considered the LR fuzzy linear system (FLRLS) of the form

(6) $$\begin{array}{c} A\tilde{x}=\tilde{b}, \end{array}$$

where *A* is a *m* × *n* crisp matrix, the unknown vector $\tilde{x}={\left({\tilde{x}}_{1},\ldots ,{\tilde{x}}_{n}\right)}^{T}$ consists of *n* fuzzy numbers, and the constant $\tilde{b}={\left({\tilde{b}}_{1},\ldots ,{\tilde{b}}_{m}\right)}^{T}$ is a vector consisting of *n* LR fuzzy numbers. They convert the LR fuzzy linear system (6) into the corresponding crisp linear system

(7) $$\begin{array}{c} Ax=b, \end{array}$$

and the constrained least-square problem

(8) $$\begin{array}{c} \left(\begin{array}{cc} B^{+} & -B^{-}\\ -B^{-} & B^{+} \end{array}\right)\left(\begin{array}{c} \alpha \\ \beta \end{array}\right)=\left(\begin{array}{c} b^{l}\\ b^{r} \end{array}\right),\;\left(\begin{array}{c} \alpha \\ \beta \end{array}\right)>0, \end{array}$$

where [*B* ^+^] and [*B* ^−^] are determined as follows:

(9) $$\begin{array}{c} {\left[B^{+}\right]}_{ij}=\{\begin{array}{cc} a_{ij}, & a_{ij}\geq 0,\\ 0, & a_{ij}<0, \end{array}\;\;{\left[B^{-}\right]}_{ij}=\{\begin{array}{cc} a_{ij}, & a_{ij}<0,\\ 0, & a_{ij}\geq 0, \end{array} \end{array}$$

for all *i* = 1,…, *m* and *j* = 1,…, *n*. Also, let *x* = (*x*_1_,…, *x*_*n*_)^*T*^, *b* = (*b*_1_,…, *b*_*m*_)^*T*^, *α* = (*α*_1_,…, *α*_*n*_)^*T*^, *β* = (*β*_1_,…, *β*_*n*_)^*T*^, *b* ^*l*^ = (*b*_1_^*l*^,…, *b*_*m*_^*l*^)^*T*^, and *b* ^*r*^ = (*b*_1_^*r*^,…, *b*_*m*_^*r*^)^*T*^. They define

(10) $$\begin{array}{c} r_{\tilde{x}}={||B^{+}\alpha -B^{-}\beta -b^{l}||}_{2}^{}+{||-B^{-}\alpha +B^{+}\beta -b^{r}||}_{2}^{}. \end{array}$$

For solving FLRLS (6), they must solve systems (7) and (8).

- If the crisp system (7) lacks solution, then the FLRLS (6) does not have any solution.
- If *x* is a solution of the system *Ax* = *b* and *α*, *β* are the solutions of the constrained least-square problem (8), then the corresponding $\tilde{x}={\left(x,\alpha ,\beta \right)}_{\text{LR}}$ is said to be an exact solution of (6).
- If $r_{\tilde{x}}>0$ for all $\tilde{x}\in F{\left(R^{n}\right)}_{\text{LR}}$, then the system (10) lacks a solution, and hence FLRLS (6) lacks an exact solution. In this case, if $\tilde{x}={\left(x,\alpha ,\beta \right)}_{\text{LR}}$ is a solution of the system *Ax* = *b* and *α*, *β* are the solutions of the constrained least-square problem (8).

This method [24] is applicable when in the crisp linear system (7) *A* ∈ *R* ^*m*×*n*^ and rank⁡(*A*) = *m*.

**Example 8**

Consider the LR fuzzy linear system

(11) $$\begin{array}{c} {\tilde{x}}_{1}-{\tilde{x}}_{2}={\left(1,1,1\right)}_{\text{LR}},\\ {\tilde{x}}_{1}+3{\tilde{x}}_{2}={\left(5,1,2\right)}_{\text{LR}}. \end{array}$$

By Ghanbari's method, we obtain an exact solution ${\tilde{x}}_{1}=(2,0.625,0.875)$ using ABS algorithm and ${\tilde{x}}_{2}=(1,0.125,0.375)$ using* lsqlin* function of MATLAB software.

(2) Allahviranloo et al. [27] transform the LR fuzzy linear system

(12) $$\begin{array}{c} A\left(x,\alpha_{x},\beta_{x}\right)=\left(b,\alpha_{b},\beta_{b}\right) \end{array}$$

into the 1-level crisp linear system (7) and the following minimization problem:

(13) min⁡z1+z2s.t.−z1≤∑aij>0aijαxj−∑aij<0aijβxj−αbi≤z1,                i=1,…,n,−z2≤∑aij>0aijβxj−∑aij<0aijαxj−βbi≤z2,                i=1,…,n,     αxj,βxj,z1,z2≥0, j=1,…,n.

The spreads of fuzzy number vector solution are obtained from the model (13). Finally, by allocating these spreads to vector solution of 1-level system, the fuzzy number vector solution results and if the computed solution satisfies the FLS, it can be as exact or approximated solution.

- If the crisp system (7) lacks solution, then the FLRLS (12) does not have any solution.
- If model (13) has more than two optimal solutions then it has a set of solutions.

**Example 9**

Consider the LR fuzzy linear system

(14) $$\begin{array}{c} {\tilde{x}}_{1}-{\tilde{x}}_{2}={\left(1,2,3\right)}_{\text{LR}},\\ {\tilde{x}}_{1}+{\tilde{x}}_{2}={\left(3,3,2\right)}_{\text{LR}}. \end{array}$$

Using Allahviranloo's method, the vector solution of the 1-level system is (2,1)^*t*^. This fuzzy linear system has infinite number of fuzzy number vector solutions as follows:

(15) $$\begin{array}{c} \left(\begin{array}{c} {\tilde{x}}_{1}\\ {\tilde{x}}_{2} \end{array}\right)=\left(\begin{array}{c} \lambda \left(2,1,1\right)+\left(1-\lambda \right)\left(2,2,2\right)\\ \lambda \left(1,2,1\right)+\left(1-\lambda \right)\left(1,1,0\right) \end{array}\right),\;\lambda \in \left[0,1\right]. \end{array}$$

The existing methods are incapable of finding the minimal solution for the following LR fuzzy linear system:

(16) $$\begin{array}{c} {\tilde{x}}_{1}⊕\left(-1\right){\tilde{x}}_{2}={\left(1,1,1\right)}_{\text{LR}},\\ 2{\tilde{x}}_{1}⊕\left(-2\right){\tilde{x}}_{2}={\left(2,2,2\right)}_{\text{LR}}. \end{array}$$

### 3.2. Singular LR Fuzzy Linear Systems

In this subsection, the singular consistent or inconsistent LR fuzzy linear systems is introduced.

**Definition 10**

The *n* × *n* linear system

(17) $$\begin{array}{c} \left(\begin{array}{ccc} a_{11} & \cdots & a_{1n}\\ \vdots & \ddots & \vdots \\ a_{n1} & \cdots & a_{nn} \end{array}\right)\left(\begin{array}{c} {\tilde{x}}_{1}\\ \vdots \\ {\tilde{x}}_{n} \end{array}\right)=\left(\begin{array}{c} {\tilde{b}}_{1}\\ \vdots \\ {\tilde{b}}_{n} \end{array}\right), \end{array}$$

where the *n* × *n* crisp singular matrix *A* = (*a*_*ij*_) and LR fuzzy vector $\tilde{b}$ are given and $\tilde{x}=F{\left(R^{n}\right)}_{\text{LR}}$ is an unknown LR fuzzy vector to be found, is called a singular LR fuzzy system of equations (SFLRLS).

In Definition 10, let ${\tilde{x}}_{i}=(x_{i},\alpha_{x_{i}},\beta_{x_{i}})$ and ${\tilde{b}}_{i}=\left(b_{i},\alpha_{b_{i}},\beta_{b_{i}}\right)$,1 ≤ *i* ≤ *n*. The *n* × *n* singular LR fuzzy system of (17) can be extended into the 3*n* × 3*n* crisp linear system

(18) $$\begin{array}{c} \left(\begin{array}{ccc} A & 0 & 0\\ 0 & B & C\\ 0 & C & B \end{array}\right)\left(\begin{array}{c} x\\ x_{\alpha }\\ x_{\beta } \end{array}\right)=\left(\begin{array}{c} b\\ b_{\alpha }\\ b_{\beta } \end{array}\right),\\ \text{abbreviated}\;\;\text{as}\;\;SX=Y, \end{array}$$

wherein

(19) $$\begin{array}{c} {\left[B\right]}_{ij}=\{\begin{array}{cc} a_{ij}, & a_{ij}\geq 0,\\ 0, & a_{ij}<0, \end{array}\;\;{\left[C\right]}_{ij}=\{\begin{array}{cc} a_{ij}, & a_{ij}<0,\\ 0, & a_{ij}\geq 0, \end{array}\\ x_{\alpha }=\left(\begin{array}{c} \alpha_{x_{1}}\\ \vdots \\ \alpha_{x_{n}} \end{array}\right),\;\;x_{\beta }=\left(\begin{array}{c} \beta_{x_{1}}\\ \vdots \\ \beta_{x_{n}} \end{array}\right),\\ b_{\alpha }=\left(\begin{array}{c} \alpha_{b_{1}}\\ \vdots \\ \alpha_{b_{n}} \end{array}\right),\;\;b_{\beta }=\left(\begin{array}{c} \beta_{b_{1}}\\ \vdots \\ \beta_{b_{n}} \end{array}\right). \end{array}$$

**Definition 11**

The singular LR fuzzy linear system (17) is called a singular consistent LR fuzzy linear system while the systems (18) are consistent crisp linear systems, that is, rank [*S*] = rank [*S* | *Y*], inconsistent otherwise.

## 4. Minimal Solution of Singular LR Fuzzy Linear Systems

Let

(20) $$\begin{array}{c} A\tilde{x}=\tilde{b} \end{array}$$

be a singular consistent LR fuzzy linear system and let (18) be the extended crisp linear system of it. The coefficient matrix system (18) is a singular matrix [16, 32]. In this section, the capability of generalized inverses in finding minimal solution of the singular consistent LR fuzzy linear system (20) is investigated. Equivalent systems of linear equations have exactly the same solutions [33]. Therefore, the fundamental theorem for the singular consistent LR fuzzy linear system is given.

**Theorem 12 (fundamental theorem)**

The system (20) is equivalent to the singular consistent crisp linear system

(21) $$\begin{array}{c} Ax=b,\;\;k=\text{ ind }\left(A\right), \end{array}$$

and the singular consistent constrained linear system

(22) $$\begin{array}{c} \left(\begin{array}{cc} B & C\\ C & B \end{array}\right)\left(\begin{array}{c} x_{\alpha }\\ x_{\beta } \end{array}\right)=\left(\begin{array}{c} b_{\alpha }\\ b_{\beta } \end{array}\right),\;\left(\begin{array}{c} x_{\alpha }\\ x_{\beta } \end{array}\right)>0. \end{array}$$

### 4.1. Notes

**Note 1**

If model (22) has a solution, then (20) has an exact LR fuzzy solution and (22) gives exact solutions; otherwise we get a set of approximated solutions. For computing approximated solution instead of the singular constrained linear system (22), we solve the following singular constrained linear system:

(23) $$\begin{array}{c} \left(\begin{array}{cc} B & C\\ C & B \end{array}\right)\left(\begin{array}{c} x_{\alpha }\\ x_{\beta } \end{array}\right)=\left(\begin{array}{c} b_{\alpha }\\ b_{\beta } \end{array}\right),\;\left(\begin{array}{c} x_{\alpha }\\ x_{\beta } \end{array}\right)\geq ɛe, \end{array}$$

where *e* = (1,…, 1)^*T*^ ∈ *R* ^*n*^ and *ɛ* > 0 is a user-defined parameter.

**Note 2**

If model (22) has a set of solutions and (21) is a consistent system, then (20) has a set of LR fuzzy exact solutions.

**Note 3**

If the crisp linear system (21) is a singular inconsistent linear system, the singular LR fuzzy linear system (20) has no solution.

**Note 4**

We observe that (*x*, *x*_*α*_, *x*_*β*_) is the exact minimal LR fuzzy solution of the singular consistent LR fuzzy linear system (20) if and only if *x* is a minimal LR fuzzy solution of the singular consistent crisp linear system (21) and the following constrained least-square problem:

(24) z=min⁡{||Bxα−Cxβ−bα||2+||−Bxα+Cxβ−bβ||2}s.t. xα>0,   xβ>0,

has a solution with Z* = 0; otherwise, the singular consistent LR fuzzy linear system has a set of approximated minimal LR fuzzy solutions.

### 4.2. Solution Strategy

According to Theorem 12, for solving (20) we must solve the system (21) and singular constrained linear system (22). Finding positive solutions for linear systems of equations has been investigated [34]. Nonnegativity of matrices and their group or Drazin [35] inverses and pseudoinverse [36] were investigated.

#### 4.2.1. Solving Singular Crisp Linear Systems (21)

**Theorem 13 (see [20])**

*A* ^*D*^ *b* is a solution of (21) if and only if *b* ∈ range(*A* ^*k*^), and *A* ^*D*^ *b* is an unique solution of (21) provided that *x* ∈ range(*A* ^*k*^).

**Theorem 14 (see [37])**

The minimal solution of the system (21) is *x* = *A* ^+^ *b*.

**Theorem 15 (see [28])**

Let *A* ∈ *C* ^*n*×*n*^, *b* ∈ *C* ^*n*^. Then (21) is consistent if and only if, for some *A* ^(1)^,

(25) $$\begin{array}{c} AA^{\left(1\right)}b=b, \end{array}$$

in which case the general solution of (21) is

(26) $$\begin{array}{c} x=A^{\left(1\right)}b+\left(I-A^{\left(1\right)}A\right)y, \end{array}$$

for arbitrary *y* ∈ *C* ^*n*^.

#### 4.2.2. Solving Singular Constrained Linear System (22)

Illustrative examples for finding positive exact solution for singular linear systems,

(27) $$\begin{array}{c} \left(\begin{array}{cc} B & C\\ C & B \end{array}\right)\left(\begin{array}{c} x_{\alpha }\\ x_{\beta } \end{array}\right)=\left(\begin{array}{c} b_{\alpha }\\ b_{\beta } \end{array}\right), \end{array}$$

using generalized inverses are given in the next section. It is possible that the generalized inverses do not obtain the positive solution for the system (27). In this case, we use the* lsqlin* function of MATLAB software. The capability of the* lsqlin* function of MATLAB software in finding exact or approximated solutions is illustrated in next the section.

## 5. Numerical Results

In this section, solving singular LR fuzzy linear systems is illustrated.

**Example 16**

Consider the following singular consistent LR fuzzy linear system:

(28) $$\begin{array}{c} {\tilde{x}}_{1}⊕{\left(-1\right)\tilde{x}}_{2}={\left(1,1,1\right)}_{\text{LR}},\\ 2{\tilde{x}}_{1}⊕\left(-2\right){\tilde{x}}_{2}={\left(2,2,2\right)}_{\text{LR}}. \end{array}$$

According to Tables 1 and 2 Drazin inverse, pseudoinverse, a {1}-inverse and the* lsqlin* function gives a set of exact LR fuzzy solution for (28).

The exact minimal LR fuzzy solution of the system (28) is ${\tilde{x}}_{1},{\tilde{x}}_{2}=(0.5000,-0.5000,0.5000)$.

**Example 17**

Consider the following singular consistent LR fuzzy linear system:

(29) $$\begin{array}{c} \left(\begin{array}{ccc} 1 & -1 & -1\\ 1 & -4 & 4\\ 3 & -3 & -3 \end{array}\right)\left(\begin{array}{c} {\tilde{x}}_{1}\\ {\tilde{x}}_{2}\\ {\tilde{x}}_{3} \end{array}\right)=\left(\begin{array}{c} {\left(5,1,2\right)}_{\text{LR}}\\ {\left(-2,10,1\right)}_{\text{LR}}\\ {\left(15,3,6\right)}_{\text{LR}} \end{array}\right). \end{array}$$

The solutions obtained by generalized inverses for the system (29) are listed in Tables 3 and 4.

The generalized inverses are incapable of finding an exact LR fuzzy solution for (29). Therefore, we use the* lsqlin* function of MATLAB software for finding a set of approximated solutions (see Table 5).

The approximated minimal LR fuzzy solution with *ɛ* = 10^−3^ is

(30) $$\begin{array}{c} {\tilde{x}}_{1}=\left(1.5918,0.1631,0.1703\right),\\ {\tilde{x}}_{2}=\left(-1.2551,0.0941,0.7236\right),\\ {\tilde{x}}_{3}=\left(-2.1531,1.7356,0.1133\right). \end{array}$$

**Example 18**

Now consider a system where the coefficient matrix is the same as in the previous system, but with the LR fuzzy vector on the right-hand side leads to an exact minimal LR fuzzy solution

(31) $$\begin{array}{c} \left(\begin{array}{ccc} 1 & -1 & -1\\ 1 & -4 & 4\\ 3 & -3 & -3 \end{array}\right)\left(\begin{array}{c} {\tilde{x}}_{1}\\ {\tilde{x}}_{2}\\ {\tilde{x}}_{3} \end{array}\right)=\left(\begin{array}{c} {\left(1,1,1\right)}_{\text{LR}}\\ {\left(-1,3,3.25\right)}_{\text{LR}}\\ {\left(3,3,3\right)}_{\text{LR}} \end{array}\right). \end{array}$$

According to Tables 6 and 7, the system (31) has a set of the exact minimal LR fuzzy solutions.

Hence for *λ* ∈ [0,1], the singular LR fuzzy linear system (31) has a set of exact minimal LR fuzzy solution as follows:

(32) $$\begin{array}{c} \left(\begin{array}{c} {\tilde{x}}_{1}\\ {\tilde{x}}_{2}\\ {\tilde{x}}_{3} \end{array}\right)=\left(\begin{array}{c} \lambda {\left(0.3061,0.2885,0.2948\right)}_{\text{LR}}+\left(1-\lambda \right){\left(0.3061,0.2891,0.2942\right)}_{\text{LR}}\\ \lambda {\left(-0.1837,0.3638,0.3365\right)}_{\text{LR}}+\left(1-\lambda \right){\left(-0.1837,0.3682,0.3401\right)}_{\text{LR}}\\ \lambda {\left(-0.5102,0.3414,0.3750\right)}_{\text{LR}}+\left(1-\lambda \right){\left(-0.5102,0.3376,0.3707\right)}_{\text{LR}} \end{array}\right). \end{array}$$

**Example 19 (singular dual LR fuzzy linear systems)**

Usually, there is no inverse element for an arbitrary LR fuzzy number [38]. Also, if $\tilde{u}=F{\left(R^{1}\right)}_{\text{LR}}$ is an arbitrary LR fuzzy number, then we have

(33) $$\begin{array}{c} \tilde{u}⊕-\tilde{u}\neq 0. \end{array}$$

Therefore, the following two fuzzy linear systems are not equivalent

(34) $$\begin{array}{c} A_{1}\tilde{x}+b_{1}=A_{2}\tilde{x}+b_{2},\;\;\left(A_{1}-A_{2}\right)\tilde{x}=\left(b_{2}-b_{1}\right). \end{array}$$

In [38] the necessary and sufficient conditions for $A_{1}\tilde{x}=A_{2}\tilde{x}+{\tilde{b}}_{2}$ were given, where *A*_1_ and *A*_2_ are square matrices and in [25] on the DFLRLS (17) was discussed. Now, we consider an economic application of minimal solution of general dual fuzzy linear systems. The market price of goods and the quantity produced are determined by the equality between supply and demand. Suppose that demand and supply are linear functions of the price

(35) $$\begin{array}{c} q_{d}+a=b*p+c,\\ q_{s}+d=e*p+f, \end{array}$$

where *q*_*s*_ is the quantity supplied, which is required to be equal to *q*_*d*_, the quantity requested, *p* is the price, *a*, *b*, *c*, *d*, *e*, and *f* are coefficients to be estimated, where the coefficients *a*, *c*, *d*, and *f* are represented by LR fuzzy numbers, and *b* and *e* are crisp numbers. By imposing the equality between quantity supplied and requested, the following general dual fuzzy linear system should be solved:

(36) $$\begin{array}{c} {\tilde{x}}_{1}⊕{\left(5,5,4.5\right)}_{\text{LR}}={\tilde{x}}_{2}⊕{\left(1,1,1\right)}_{\text{LR}},\\ 2{\tilde{x}}_{1}⊕{\left(10,10,9\right)}_{\text{LR}}=2{\tilde{x}}_{2}⊕{\left(2,2,2\right)}_{\text{LR}}. \end{array}$$

Using Drazin inverse we get the exact solution ${\tilde{x}}_{1}={\left(4,4,3.5\right)}_{\text{LR}}$ and ${\tilde{x}}_{2}={\left(8,8,7\right)}_{\text{LR}}$. For economic analysis, we refer to [17, 39].

## 6. Conclusions and Suggestions

In this paper, the *n* × *n* singular LR fuzzy linear system $A\tilde{x}=\tilde{b}$ is transformed into the 3*n* × 3*n* singular crisp linear system *SX* = *Y*. Such systems are divided into two parts: singular consistent LR fuzzy linear systems and singular inconsistent LR fuzzy linear systems. The singular consistent LR fuzzy linear system $A\tilde{x}=\tilde{b}$ has an exact minimal LR fuzzy solution or a set of the approximated minimal LR fuzzy solutions. It is possible that the generalized inverses be incapable of finding the exact or approximated minimal LR fuzzy solution of the system $A\tilde{x}=\tilde{b}$. In this case, we use the* lsqlin* function of MATLAB software. We hope that this paper will be a new path to improve solving singular consistent LR fuzzy linear systems using preconditioning techniques and iterative methods.

## Figures and Tables

**Table 1:** Finding the left and right spreads.

Using	*α*_*x*_1__	*α*_*x*_2__	*β*_*x*_1__	*β*_*x*_2__	||(*x*_*α*_, *x*_*β*_)^*t*^||_2_
Drazin inverse	0.3333	0.6667	0.3333	0.6667	1.0541
Pseudoinverse	0.5000	0.5000	0.5000	0.5000	1.0000
{1}-inverse	0.5000	0.5000	0.5000	0.5000	1.0000
Other {1}-inverse	2.0000	2.0000	−1.0000	−1.0000	3.1623
*lsqlin* function	0.5000	0.5000	0.5000	0.5000	1.0000

**Table 2:** Finding the mean values.

Using	*x*_1_	*x*_2_	||*x*||_2_
Drazin inverse	−1.0000	−2.0000	2.2361
Pseudoinverse	0.5000	−0.5000	0.7071
{1}-inverse	0	−1.0000	1.0000

**Table 3:** Finding the mean values.

Using	*x*_1_	*x*_2_	*x*_3_	||*x*||_2_
Drazin inverse	−1.0476	−2.9048	−3.1429	4.4060
Pseudoinverse	1.5918	−1.2551	−2.1531	2.9572
{1}-inverse	4.6667	0.6667	−1.0000	4.8189

**Table 4:** Finding the left and right spreads.

Using	*α*_*x*_1__	*α*_*x*_2__	*α*_*x*_3__	*β*_*x*_1__	*β*_*x*_2__	*β*_*x*_3__	||(*x*_*α*_, *x*_*β*_)^*t*^||_2_
Drazin inverse	0.4762	0.7143	1.4286	−0.1429	0.9524	−0.4286	1.9720
Pseudoinverse	0.0952	0.3095	1.4524	0.2381	1.0238	−0.1190	1.8257
{1}-inverse	−1.0000	−1.0833	1.7500	1.3333	1.0000	1.0000	3.0023

**Table 5:** Finding the left and right spreads.

Using	*α*_*x*_1__	*α*_*x*_2__	*α*_*x*_3__	*β*_*x*_1__	*β*_*x*_2__	*β*_*x*_3__	*ε*
*lsqlin* function	0.1631	0.0941	1.7356	0.1703	0.7236	0.1133	10^−3^
*lsqlin* function	0.1793	0.1000	1.7460	0.1540	0.7092	0.1115	10^−2^
*lsqlin* function	0.1867	0.1006	1.7528	0.1466	0.7005	0.1128	10^−1^

**Table 6:** Finding the left and right spreads.

Using	*α*_*x*_1__	*α*_*x*_2__	*α*_*x*_3__	*β*_*x*_1__	*β*_*x*_2__	*β*_*x*_3__	||(*x*_*α*_,*x*_*β*_)^*t*^||_2_
Drazin inverse	0.2857	−0.1548	0.8571	0.2976	−0.1786	0.8929	1.3259
Pseudoinverse	0.2891	0.3682	0.3376	0.2942	0.3401	0.3707	0.8203
{1}-inverse	−1.0000	−1.0833	1.7500	1.3333	1.0000	1.0000	3.0023
*lsqlin* function	0.2885	0.3638	0.3414	0.2948	0.3365	0.3750	0.8203

**Table 7:** Finding the mean values.

Using	*x*_1_	*x*_2_	*x*_3_	||*x*||_2_
Drazin inverse	−0.2381	−0.5238	−0.7143	0.9172
Pseudoinverse	0.3061	−0.1837	−0.5102	0.6227
{1}-inverse	−1.0000	−1.0000	−1.0000	1.7321
